# Resistance to targeted therapies: delving into FLT3 and IDH

**DOI:** 10.1038/s41408-022-00687-5

**Published:** 2022-06-09

**Authors:** Sai Prasad Desikan, Naval Daver, Courtney DiNardo, Tapan Kadia, Marina Konopleva, Farhad Ravandi

**Affiliations:** Departments of Leukemia, The University of Texas MD Anderson Cancer Center, 1400 Holcombe Boulevard, Houston, TX USA

**Keywords:** Acute myeloid leukaemia, Leukaemia

## Abstract

Recent advances in FLT3 and IDH targeted inhibition have improved response rates and overall survival in patients with mutations affecting these respective proteins. Despite this success, resistance mechanisms have arisen including mutations that disrupt inhibitor-target interaction, mutations impacting alternate pathways, and changes in the microenvironment. Here we review the role of these proteins in leukemogenesis, their respective inhibitors, mechanisms of resistance, and briefly ongoing studies aimed at overcoming resistance.

## Introduction

The advent of imatinib for patients with chronic myeloid leukemia set the stage for targeted therapies in other hematologic malignancies. The development of molecularly targeted inhibitors has been challenging in acute myeloid leukemia (AML) on account of the polyclonal nature of the disease and the complexity of the underlying molecular aberrations. It took 14 years from the advent of Imatinib to the approval of the first molecularly targeted agent in AML, midostaurin. However, since then several agents have been developed targeting a variety of pathways.

Despite the development of these effective agents, achieving durable remission using these drugs has been challenging. The mechanisms underlying resistance to these agents are complex and are being extensively investigated.

FMS-like tyrosine kinase 3 (FLT3) inhibitors and Isocitrate dehydrogenase (IDH) inhibitors have been successfully employed in AML, inducing complete responses in a significant proportion of patients both as monotherapy and as a part of combination regimens. However, resistance to these therapies lead to relapse and progression. Increased understanding of the mechanisms of resistance can expedite the development of new agents that overcome this resistance. Here we will review mechanisms of resistance to therapies targeting FLT3 and IDH. Possible combinations designed to overcome these resistance mechanisms will also be briefly discussed.

## FLT3

**Fig. 1:**
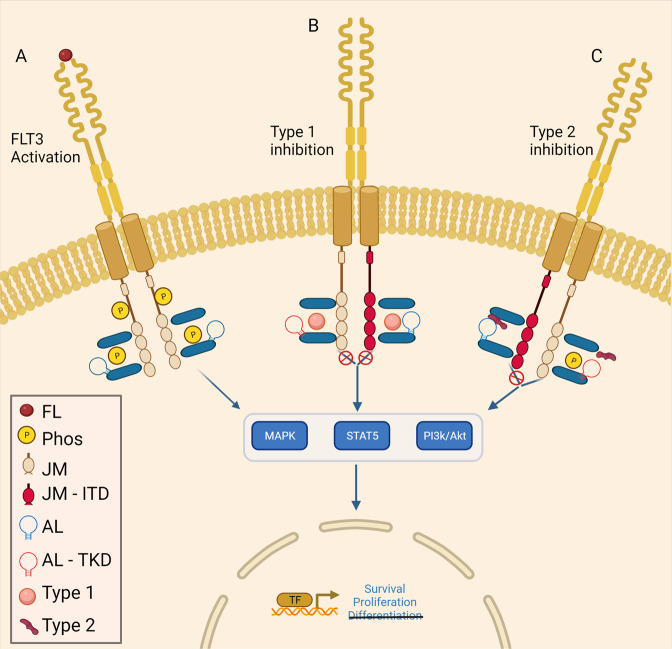
FLT3 activation and inhibition. **A** FLT3 ligand binds FLT3 receptor with phosphorylation of the juxtamembrane domain. The activation loop assuming an open configuration, resulting in the active conformation. After phosphorylation, the PI3K/Akt, the STAT5, and the MAPK pathways are activated with alterations in transcription. **B** Type 1 inhibitors bind the ATP binding side regardless of ITD (red juxtamembrane domain) or TKD (red activation loop) mutations. **C** The left side depicts type 2 inhibitor binding a protein with an ITD mutation (red juxtamembrane domain). Type 2 inhibitors bind the activation loop, stabilizing the inactive conformation. On the right side, the TKD mutation (red activation loop), shifts the activation loop into an open conformation. The type 2 inhibitor is unable to bind, permitting ATP binding. Figure created with Biorender.com.

FLT3 is a class III tyrosine kinase composed of an extracellular immunoglobulin-like domain, a transmembrane helix, a juxtamembrane (JM) domain, and a kinase domain comprised of N and C lobes along with an activation loop that sits between them [1]. FLT3 assumes 2 conformations: inactive and active. The JM domain binds the N lobe of the tyrosine kinase domain and the activation loop; thereby stabilizing the activation loop in a closed configuration and the protein in inactive conformation. Upon binding FLT3 ligand, homodimerization occurs leading to phosphorylation of the JM domains. Phosphorylation shifts the JM domain out of an autoinhibitory position. The activation loop subsequently adopts an open conformation, revealing the ATP binding site, as demonstrated in Fig. 1A [1, 2]. FLT3 signaling activates downstream pathways: PI3K/Akt, MAPK, and STAT5 [3–5].

FLT3 is expressed in CD34+ progenitor cells, dendritic cells, natural killer cells, and T cells. FLT3 in conjunction with granulocyte-macrophage colony-stimulating factor (GM-CSF) is involved in hematopoiesis and differentiation of dendritic and natural killer cells [6].

## Role in leukemogenesis

FLT3 is upregulated in a substantial fraction of AML patients with growth mediated by ligand binding [7]. However, *FLT3* mutations are observed in 30–35% of patients [8]. These include *FLT3* internal tandem duplications (ITD) mutations and *FLT3* tyrosine kinase domain (TKD) mutations. *FLT3*-ITD mutations comprise ~80% of all FLT3 mutations [8, 9].

The *FLT3* gene is located on chromosome 13. *FLT3-*ITD mutations occur on exon 11, between codons 590 and 600. These mutations alter the length of JM domain; thereby disrupting the autoinhibitory interaction between the JM domain and the N lobe. While the length of tandem duplications does not impact prognosis, the positioning and charge at R595 is vital for survival of the clone [10]. *FLT3*-TKD mutations occur at exon 17, codon 835 [9]. These point mutations result in amino acid changes in the activation loop. With these mutations, the protein remains in the active conformation with the activation loop in an open configuration [6, 11].

The prognosis of *FLT3-*ITD mutations is determined by the allelic ratio and co-mutation with *NPM1*. In the European LeukemiaNet stratification of AML, *FLT3-*ITD mutations with allelic ratios of *FLT3-*ITD to *FLT3-*WT less than 0.5 with a concomitant *NPM1* are favorable, *FLT3-*ITD mutations with allelic ratios of *FLT3-*ITD to *FLT3-*WT <0.5 without a concomitant *NPM1* or *FLT3-*ITD with high (*FLT3-*ITD^high^) allelic ratios (>0.5) with a concomitant *NPM1* mutation are intermediate risk, and *FLT3-*ITD^high^ with wild type *NPM1* is considered adverse risk [12]. *FLT3-*ITD^high^ is independently associated with decreased overall survival (OS) and disease-free survival (DFS) [9].

Singularly*, FLT-*TKD mutations do not confer decreased survival in patients with AML; however, there are trends to decreased overall survival in patients with a co-occurring *FLT3-*ITD or a co-occurring *MLL-*partial tandem duplication mutations, as demonstrated by Bacher et al. [13].

## FLT3 inhibitors

Inhibitors targeting FLT3 are categorized by the conformation they bind and their specificity, as depicted in Table 1. Type 1 inhibitors inhibit the ATP binding pocket and are not dependent on conformation, as demonstrated in Fig. 1B [14]. Type 2 inhibitors stabilize FLT3 in the inactive conformation with the activation loop in a closed configuration (phenyalanine in the DFG motif protruding into the hydrophobic groove). As *FLT3-*TKD mutations disrupt the activation loop, maintaining an open configuration, Type 2 inhibitors are unable to bind FLT3 in this context, as demonstrated in Fig. 1C [15]. FLT3 inhibitors are further characterized based on specificity.

**Table 1:** FLT3 inhibitors are characterized based on mechanism of inhibition and generation.

Table 1	Type 1	Type 2
First generation	Midostaurin	Sorafenib
Second generation	Giltritenib Crenolanib	Quizartinib

First-generation FLT3 inhibitors are broad kinase inhibitors [16]. This generation of inhibitors includes sorafenib and midostaurin. Sorafenib, in particular, has a broad kinase profile with the initial trials utilizing sorafenib in the relapsed/refractory setting regardless of FLT3 mutations status in part due to this broad activity. Sorafenib alone and in combination with chemotherapy demonstrated significant activity in patients with *FLT3* mutations [17]. Midostaurin was the first FLT3 inhibitor approved by the United States Food and Drug Administration (FDA) for treating patients with *FLT3*-mutated AML, in combination with chemotherapy [18]. The phase 3, CALGB10603 demonstrated a survival benefit with the addition of midostaurin to chemotherapy with a 4-year OS of 51% as compared to chemotherapy at 44% [19].

Second-generation Type 1 (gilteritinib and crenolanib) and Type 2 (quizartinib) inhibitors are more specific for FLT3 kinase. Gilteritinib has been approved by the FDA, for patients with relapsed *FLT3* (ITD and TKD) mutated AML [20]. Monotherapy with gilteritinib and quizartinib has shown superior response rates in *FLT3* mutant relapsed/refractory AML when compared to standard salvage chemotherapy as demonstrated by the phase 3 ADMIRAL and QuANTUM-R trials, respectively [21, 22].

Regardless of type, FLT3 inhibitor monotherapy in patients with *FLT3-*ITD mutations induces responses in 2 distinct patterns: differentiation in which mature myeloid cells with *FLT3-*ITD mutations can be detected and cytotoxicity in which a hypocellular marrow is initially present, usually resulting in lower *FLT3*-ITD VAF as compared to the differentiation pattern [23, 24].

## Resistance mechanisms

Despite the activity of FLT3 inhibition in the frontline and relapsed/refractory disease, 30–45% of patients relapse on therapy [25–27]. Resistance occurs through a variety of mechanisms: *FLT3* mutations, secondary mutations impacting other pathways, and factors within the micro-environment. These processes are not mutually exclusive and co-occur frequently [28].

### FLT3 mutations

Type 2 inhibitors maintain the inactive, closed-loop conformation. Secondary *FLT3-*TKD mutations alter the activation loop, occurring in ~30% of patients at the time of progression on treatment with type 2 FLT3 inhibitor-based therapies [29]. The majority of these mutations target the D835(D835V, D835F, and D835Y) residue. A variety of subclones can exist with: *FLT3-*TKD without *FLT3* ITD, *FLT3* -ITD without *FLT3-*TKD mutations, and clones with multiple *FLT3-*TKD mutations [30]. While most patients who develop resistance to therapy with TKD mutations do so after treatment, clonal evolution with TKD mutated subclones does occur as illustrated by Baker et al. [28].

TKD mutations affecting the activation loop are uncommon mechanisms of resistance in patients on Type 1 inhibitors. However, mutations in other areas including F691L (gatekeeper mutation within the active site), Y693C, Y693N, and D698N may still confer resistance to Type 1 inhibitors [26, 31]. These mutations alter binding to the active site with resulting residue changes affecting side chains, aromaticity, direct steric clash, and indirect steric clash [31, 32].

While uncommon, ITD mutations can also occur within the kinase domains, as demonstrated by Breitenbuecher et al. in a patient treated with midostaurin [33]. Clonal evolution that selects for certain *FLT3*-mutated populations can occur leading to the expansion of FLT3 inhibitor-resistant subclones. Increased MCL1, persistent ERK activation, and increased STAT3 phosphorylation were noted in these patients. STAT3 and MCL1 inhibition resulted in response to therapy [33].

### Secondary mutations in other pathways

Mutations impacting parallel pro-survival pathways are common mechanisms of resistance with targeted therapies. In regard to FLT3 inhibitors, a diverse range of mutations are utilized by leukemic clones in order to promote resistance.

RAS pathway mutations are the most common mutation-derived mechanism of resistance to type 1 inhibitors, occurring in ~30% of patients who relapse after having achieved a remission to type 1 inhibitors. These mutations can occur as new mutations after treatment or as clonal expansion with increasing variant allele frequency (VAF) throughout the treatment course. Higher variant allele frequencies in RAS/MAPK mutations are associated with poorer outcomes in both primary and secondary relapse settings. Compared to type 1 inhibitors, RAS pathway mutations occur less frequently with type 2 inhibition, occurring in only 6% of patients relapsing post type 2 inhibitors [34]. While *FLT3-*TKD mutations are the predominant mechanism of resistance with type 2 inhibitors, single-cell DNA sequencing in patients receiving quizartinib showed *RAS*-mutated clones can expand independent of *FLT3* mutations or concomitantly with *FLT3-*TKD mutations [35].

A myriad of other mutations contributes to resistance. In one study investigating resistance to crenolanib, the VAFs of various mutations were monitored prior to the initiation of therapy until relapse. Germline *TET2, RUNX1, U2AF1, DNMT3A, IDH1*, and *SF3B1* were noted to be pre-existing with VAFs in some patients 50% or more. Notably, the VAFs remained constant throughout therapy, suggesting primary resistance in the founder clone, despite FLT3 inhibition. Other mutations such as *ASXL1, BCOR, STAG2*, and *CEBPA* had an increase in VAF suggesting that these mutations could be contributing to resistance [26]. Parallel tyrosine kinase pathways can also be utilized for resistance with JAK and PI3K/AKT known to be associated with resistance to gilteritinib, sorafenib, and midostaurin [36, 37].

### The microenvironment and cytokines

The bone marrow environment plays an important role in the preservation of *FLT3* mutated clones. Preservation of leukemic clones can be mediated by cytokines and growth factors released from the bone marrow microenvironment. Interactions between stromal cells and leukemic cells also contribute to resistance [38].

Cytokines such as CCL5 (receptor CCR5) and receptors such as CXCR4 (ligand CXCL12) play important roles in stem cell localization and survival. Elevated levels of CCL5 and the expression of CXCR4 on leukemic cells result in downstream ERK and Akt activation, promoting survival and disrupting migration of these cells to the blood. These cytokines have been shown to promote survival of FLT3 clones in a kinase-independent manner [39–42]. Furthermore, pre-clinical studies in mouse models have shown that the transcription of CXCR4 in addition to E-selectin ligand are upregulated after exposure to quizartinib, suggesting that evasion, utilizing niches within the bone marrow microenvironment, is an important component to resistance [43].

In an in vitro and in vivo study investigating the relationship between growth factors and resistance to therapy, GM-CSF resulted in activation of the RAS and the AKT pathways. In addition, GM-CSF induce activation of PIMs, anti-apoptotic kinases, in a JAK2 dependent manner. The administration of PIM and JAK2 inhibitors in combination with FLT3 inhibitors resulted in decreased survival of previously resistant clones [44]. Similarly, in another in vitro study utilizing quizartinib, FGF2 (a growth factor produced by the stroma) promoted survival of leukemic cells. FGF2 was expressed primarily by the stroma and peaked early in resistance, declining thereafter. Upon binding FGF2, leukemia cells will activate FGFR1 and by extension the MAPK pathway. Interestingly, removing FGF2 or FLT3 ligand-mediated signaling resulted in secondary mutations or clonal evolution of cells with RAS/MAPK mutations [45].

The microenvironment is dynamic and changes with therapy. FLT3 ligand in relapsed patients after intensive chemotherapy was found to be significantly higher [46]. FLT3 ligand binds FLT3-WT activating ERK, AKT, and downregulating pro-apoptotic proteins. The activation of these pathways via FLT3-WT promotes survival despite FLT-ITD inhibition [47].

Taken together, the microenvironment plays an important role in the preservation of leukemic cells via activation of alternative pathways involved in survival and apoptosis. Based on the cytokine profile in the marrow, diverse subclones can exist dependent to varying degrees on cytokines in the environment.

## Overcoming resistance

As a result of the diverse mechanisms of resistance to FLT3 inhibitors, a multitude of agents targeting downstream pathways have been studied. A phase 1 study utilizing pacritinib (a JAK and FLT3 inhibitor) in combination with chemotherapy demonstrated limited effectiveness with 3 of 13 patients responding [48]. In vivo studies have shown improved survival in mouse models with the combination of FLT3 inhibitors with MEK inhibitors, JAK inhibitors, and dasatinib (a multi-kinase inhibitor targeting BCR-ABL, c-KIT, and Src kinases) [49, 50].

FLT3 inhibitors induce remissions in two patterns: differentiation and cytotoxic with differentiation associated with higher residual VAFs [23, 24]. Combination with pro-apoptotic agents such as venetoclax, may improve survival and response by increasing the cytotoxic activity [51]. Pre-clinical models with a FLT3 inhibitor and venetoclax demonstrated synergy even in samples with resistance to FLT3 inhibitor monotherapy [52, 53]. Results from the ongoing phase 1b trial with venetoclax and gilteritinib combination therapy have yielded promising results in the relapsed or refractory setting with a composite response rate of 78 and 60% in patients with prior tyrosine kinase inhibitor therapy and prior venetoclax, respectively [54].

The most promising results have been with the triplet therapy: a FLT3 inhibitor, venetoclax, and a hypomethylating agent. In a report by Maiti et al., combination of venetoclax, decitabine, and a FLT3 inhibitor of clinician’s choice in the relapse or refractory setting yielded a composite response rate of 63% in patients who had prior tyrosine kinase inhibitors [55]. An ongoing phase 1–2 trial utilizing azacitidine with venetoclax and gilteritinib demonstrated an overall response rate of 67% and an overall survival of 10.5 months in relapsed patients including patients with prior transplant and prior treatment with FLT3 inhibitors [56].

Finally, therapies targeting the microenvironment are under development. Uproleselan, a E-selectin inhibitor, in particular has shown promise in AML, inducing a composite response of 41% when combined with chemotherapy in relapse or refractory setting [57]. In light of upregulation of E-selectin in patients with quizartinib exposure, combination therapy with uproleselan and a FLT3 inhibitor may overcome microenvironment induced resistance mechanisms.

## IDH

Both isocitrate dehydrogenase 1 (IDH1) and isocitrate dehydrogenase 2 (IDH2) share similar structures consisting of 2 subunits forming a homodimer with each subunit comprised of a large domain, a small domain, and a clasp domain. The active site is comprised of a combination of a large domain of one subunit with a small domain of another subunit. The clasp domain holds the unit together and plays an important role in maintaining the active site. Another site called the back cleft consists of large and small domains on one subunit. This site is important for maintaining conformation [58].

IDH enzymes function within the Krebs cycle in a 2-step process converting isocitrate to oxalosuccinate, reducing NADP+ to NADPH, followed by decarboxylation resulting in the formation of α-ketoglutarate [59]. These enzymes assume 2 conformations: an open conformation and a closed conformation. The closed conformation is required for catalytic activity [60]. In addition to its role in metabolism, IDH plays an important role in preventing and addressing oxidative damage via production of NADPH and activation of HIF1α [61].

## Leukemogenesis

Mutations affecting proteins involved in metabolism with resulting metabolic derangements is an established step in carcinogenesis. *IDH* mutations, specifically, occur in ~18% of patients with AML, in particular the elderly and in patients with normal karyotypes. While patients with pre-existing *IDH* mutations are at increased risk of developing AML, they usually require another driver mutation in order to induce transformation of pre-leukemic clones to AML [62]. As a result, they can occur early or late in the process of leukemogenesis. In one study utilizing single-cell sequencing, two clones with *IDH1* and *IDH2* mutations followed an initial *NPM1*, *DNMT3A*, or *RUNX1* mutation, a pattern that is well elucidated as these commonly occur together [63].

**Fig. 2:**
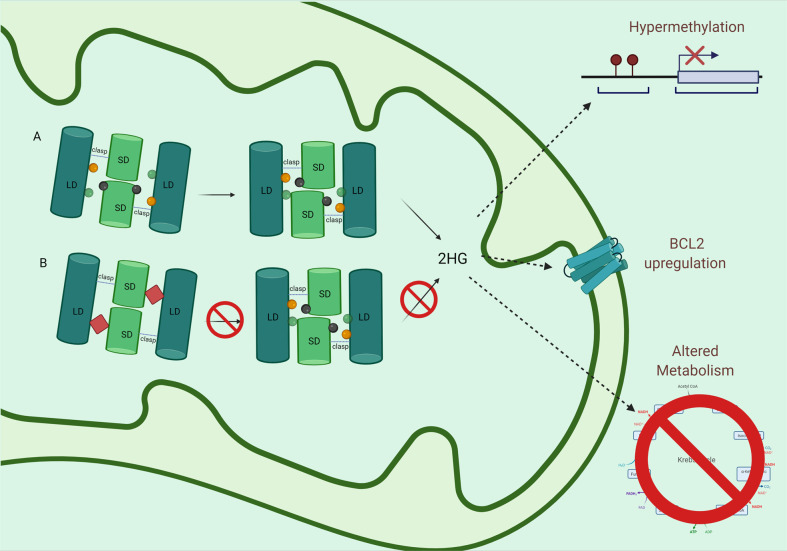
The impact of IDH mutations and IDH inhibition. **A** Mutations of IDH occur at the active site, resulting in neomorphic activity. 2HG production results in hypermethylation, increased BCL2 expression, and altered metabolism. **B** IDH inhibitors function by stabilizing the open conformation, preventing catalytic activity. Figure created with Biorender.com.

Mutations affecting *IDH* function occur in the active site: R132 in *IDH1*, R140 and R172 in *IDH2*, respectively [64]. Unlike mutations occurring in *FLT3* or other kinases that augment the underlying function, *IDH* mutations results in neomorphic enzymatic activity. As a result, IDH reduces α-ketoglutarate to 2-hydoxyglutarate (2HG), an oncometabolite which competitively inhibits aKG, as represented in Fig. 2A [65].

2HG interferes with metabolism and suppresses the Krebs cycle, decreasing the availability of α-ketoglutarate. As a result, α-ketoglutarate is generated via upregulated glutamine metabolism, a form of anapleresis, supplying carbon for both 2HG production and the Krebs cycle [66].

Independent of its function within the Krebs cycle, α-ketoglutarate binds KDM4a, TET2, and ALKBH3, enzymes important in DNA repair and methylation. 2HG binds these enzymes and inhibits them, resulting in progressive DNA damage, increased methylation (CpG island methylator phenotype), and preventing differentiation. These are thought to be the primary mechanisms by which IDH mutations contribute to leukemogenesis [65].

Finally, 2HG inhibits cytochrome C oxygenase, a component of the mitochondrial electron transport chain located in the mitochondrial membrane and an enzyme involved in addressing reactive oxygen species. While the exact underlying mechanism is unknown, inhibition results in increased expression of BCL-2. Increased BCL2 sequesters pro-apoptotic proteins, preventing apoptosis in leukemic cells [67, 68].

In summary, *IDH* mutations alter metabolism, confer resistance against differentiation, and upregulate anti-apoptotic proteins, promoting leukemogenesis.

## IDH1 and IDH2 inhibitors

IDH1 and IDH2 inhibitors both bind allosteric sites preventing their respective proteins from assuming a closed conformation required for catalytic activity. There are 2 FDA-approved IDH inhibitors: ivosidenib, an IDH1 inhibitor, and enasidenib, an IDH2 inhibitor. IDH inhibitors bind the homodimer interface, altering binding of NADPH and stabilize the open conformation of the protein (Fig. 2B). Both of these drugs result in significant reduction in the production of 2HG [69–71].

Enasidenib was the first FDA-approved IDH inhibitor, specifically in the relapsed and refractory IDH2-mutated setting. The ORR was 40.3%, CR/CRi rate was 26.8%, and a median OS of 9.3 months. The median duration of response in patient achieving CR/CRi was almost 6 months [72, 73].

The efficacy, safety, and FDA approval of ivosidenib was similarly established in a phase 1 trial of relapsed/refractory patients with *IDH1*-mutated AML. As single-agent therapy, the ORR was 41.6%, CR/CRi rate was 30%, with a median OS of 8.3 months [74]. Analysis of 34 treatment naïve patients suggested efficacy in the frontline setting with an ORR of 42.4% and a median OS of 12.6 months, leading to FDA approval for ivosidenib monotherapy for both relapsed/refractory as well as newly diagnosed, older chemotherapy-ineligible patients with IDH1-mutated AML. This trial also suggested that IDH1 mutation clearance by digital PCR (sensitivity 0.02–0.04%) was associated with increased duration of response and could be utilized as a method of MRD detection [75]. More recently, the preliminary data from the ongoing phase 3 AGILE study showed superior response and survival with the combination of ivosidenib and azacitidine compared to azacitidine with placebo with an ORR of 63% and 19% and an overall survival of 24 and 8 months, respectively [76].

## Resistance mechanisms

Leukemogenesis via *IDH* mutations is partially dependent on hypermethylation preventing differentiation. IDH inhibitors rapidly prevent 2HG production, which promotes differentiation through reversal of hypermethylation. Resistance to IDH inhibition occurs through multiple mechanisms, including 2HG rescue through second site mutations or isoform switching, preservation of a hyper-methylator phenotype through mutations in key transcription factors involved in differentiation (RUNX1, GATA2, CEBPA), as well as clonal evolution and expansion of receptor tyrosine kinase pathway mutations (i.e., KRAS, NRAS, PTPN11, FLT3). Importantly, a number of these mechanisms can coexist representing the complexity involved in the leukemic response to targeted inhibition.

### 2-hydroxyglutarate rescue

*IDH* mutations are heterozygous. Mutations conferring resistance can occur on the same allele that is affected or the opposite allele. Normally *IDH2* mutations alter amino acid expression localized to the dimer interface. Mutations occurring on the opposite allele, trans-mutations, include Q316E and I319M can confer resistance. These mutations change the structure of the enasidenib binding site on the dimer interface. As a result, enasidenib cannot bind the protein. These can also occur within the same allele, resulting in cis-mutation mediated resistance and increased 2HG [77]. Isoform switching in patients on enasidenib is rare. However, the *IDH1*-R132 has been shown to rescue 2HG production [78].

In an analysis of patients who relapsed while receiving ivosidenib, second-site *IDH1* and *IDH2* mutations were noted in 14% and 12% of patients, respectively. *IDH1* mutations cause steric interference and protein conformation changes, preventing binding of ivosidenib. *IDH2*-R140Q was the only emerging *IDH2* mutations noted. Single-cell sequencing elucidated the roles of isoform switching and *IDH2*-mutated clonal evolution. Three different patterns of *IDH2* mediated resistance exist: pre-treatment *IDH2* clones that expand, new onset *IDH2* mutations in the same clone, or *IDH2* mutations in another clone, separate from the *IDH1*-mutated clone. The resulting rescue of 2HG suggests a dependence on this pathway for survival [79].

### Altered methylation and differentiation

In a study investigating the role of leukemia stemness in IDH inhibitor resistance, two distinct clusters with differing degrees of methylation were identified in pre-treatment samples. The hypermethylated cluster had decreased response compared to more hypomethylated cluster. Hypermethylated regions included promoters for RUNX1 and other proteins involved in differentiation. Of note, both clusters had significant demethylation after therapy with the hypermethylated cluster remaining comparatively more hypermethylated after treatment.

Mutation analysis was conducted after treatment, with mutations relating to methylation occurring in 17% of samples on relapse, mainly involving *DNMT3A* and *TET2*. Mutations involving *RUNX1* and other transcription factors (*CEBPA* and *GATA2*) involved in differentiation were associated with worse prognosis.

Finally, this study details 3 patterns of resistance: RAS activation, *TET2* mutations, and *IDH1* mutations with the latter 2 associated with increased methylation at relapse, elucidating the importance of maintaining methylation and the prevention of differentiation [80].

### Clonal evolution and secondary mutations

RAS and RTK pathway mediated resistance is common to both types of inhibitors. Differentiating co-occurring mutations that grant resistance to therapy and mutations that occur after therapy impart understanding of leukemogenesis. Clonal evolution plays an essential role in mediating resistance to therapy in enasidenib-resistant patients. Expansion of co-mutated clones with FLT3, RUNX1, and RAS pathway mutations, respectively, confer a survival advantage [78]. In patients receiving ivosidenib, RAS pathway mutations were associated with lower VAF *IDH1* mutation status, suggesting a complex system in which multiple clones exist dependent to varying degrees on 2HG. In addition, it suggests the existence of clones without *IDH1* mutations. Nonetheless, response to IDH inhibition did not correlate to pre-treatment IDH VAF levels [80].

In summary, IDH inhibitor-mediated resistance is complex and dictated by a multitude of factors. Understanding 2HG rescue, the persistence of hypermethylation, and second site mutations and their interaction with each other, elucidates possible targets for therapy after IDH inhibitor resistance.

## Overcoming resistance

Multiple ongoing trial are addressing the various mechanisms of resistance to IDH inhibitors. The addition of hypomethylating agents to IDH inhibitors has improved ORR [81, 82]. Similar results have been noted with IDH inhibitor therapy with 7 + 3 [83]. Venetoclax, a BCL2 inhibitor, in combination with hypomethylating agents has been shown to be efficacious in IDH-mutated AML, with an emphasis on *IDH2*-mutated AML [84, 85]. Preliminary data from triplet therapy involving ivosidenib, venetoclax, and azacitidine appears to be promising. This combination in particular is notable due to the degree of MRD negative rates, up to 60% in both newly diagnosed and relapsed/refractory venetoclax and ivosidenib naïve patients. This combination directly addresses 2HG, the hypermethylated phenotype, upregulated anti-apoptotic mechanisms, and the polyclonal nature of IDH resistance [86]. Finally, several Pan-IDH inhibitors targeting both IDH1 and IDH2 isoforms are currently in early clinical trials [87].

## Discussion

FLT3 and IDH inhibitors represent 2 success stories in the treatment of AML, contributing to safe and efficacious regimens.

Resistance to these therapies is complicated and involves a multitude of mechanisms. For FLT3, a receptor tyrosine kinase, resistance occurs via primary mutations, secondary mutations, and changes in the microenvironment. For IDH, an enzyme that generates 2HG, resistance is dependent on 2HG rescue, changes in methylation homeostasis, clonal evolution, and secondary mutations.

The solution for resistance toward these inhibitors resides in combination regimens and novel, more potent inhibitors. Combination regimens involving these inhibitors in particular with venetoclax and hypomethylating agents have improved response and decreased resistance. Unfortunately, RAS pathway mutations continue to be a major obstacle preventing durable remissions. Further studies into the leukemogenesis before and after therapy need to be conducted in order to appropriately address these patients.

## Data Availability

As no new data was generated for this manuscript, data sharing is not applicable.
